# Machine learning to improve prognosis prediction of metastatic clear-cell renal cell carcinoma treated with cytoreductive nephrectomy and systemic therapy

**DOI:** 10.17305/bjbms.2022.8047

**Published:** 2023-05-01

**Authors:** Wenjie Yang, Lin Ma, Jie Dong, Mengchao Wei, Ruoyu Ji, Hualin Chen, Xiaoqiang Xue, Yingjie Li, Zhaoheng Jin, Weifeng Xu, Zhigang Ji

**Affiliations:** 1 Department of Urology, Peking Union Medical College Hospital, Chinese Academy of Medical Sciences and Peking Union Medical College, Dongcheng, Beijing, China

**Keywords:** Metastatic clear-cell renal cell carcinoma (mccRCC), cytoreductive nephrectomy (CN), systemic therapy, machine learning (ML), prognosis prediction model, Surveillance, Epidemiology, and End Results (SEER) database

## Abstract

Cytoreductive nephrectomy (CN) combined with systemic therapy is commonly used to treat metastatic clear-cell renal cell carcinoma (mccRCC). However, prognostic models for these patients are limited. In the present study, the clinical data of 782 mccRCC patients who received both CN and systemic therapy were obtained from the Surveillance, Epidemiology, and End Results (SEER) database (2010–2016), and patients were divided into training and internal test cohorts. A total of 144 patients who met the same criteria from our center (Peking Union Medical College Hospital) were placed in the external test cohort. The cancer-specific survival rate (CSS) at 1, 3, and 5 years was set as the research outcome. Then, four ML models, i.e., a gradient boosting machine (GBM), support vector machine (SVM), random forest (RF), and logistic regression (LR), were established. Fifteen potential independent features were included in this study. Model performance was evaluated using the area under the receiver operating characteristic curves (AUC), calibration plots, and decision curve analysis (DCA). Seven clinical features, namely, pathological grade, T stage, N stage, number of metastatic sites, brain or liver metastases, and metastasectomy, were selected for subsequent analysis via the recursive feature elimination (RFE) algorithm. In conclusion, the GBM model performed best at 1-, 3- and 5-year CSS prediction (0.836, 0.819, and 0.808, respectively, in the internal test cohort and 0.819, 0.805, and 0.786, respectively, in the external cohort). Furthermore, we divided the patients into three strata (high-, intermediate-, and low-risk) via X-tile analysis and concluded that clinically individualized treatment can be aided by these practical prognostic models.

## Introduction

Renal cell carcinoma (RCC) is one of the most common genitourinary cancers, with an increasing incidence and morbidity rate worldwide. Clear-cell RCC (ccRCC) remains the most prevalent histological subtype of renal cancer (accounting for 80%–85%). Although an ever-growing number of renal cancer patients can be detected at an early stage, 25%–30% of patients have metastasized at the time of diagnosis, and over 20% of patients will develop metastases after curative surgery, which contributes to a poor prognosis in patients [1–3].

Standard treatment strategies for metastatic ccRCC (mccRCC) have progressed substantially over the past decades. The majority of studies have shown that cytoreductive nephrectomy (CN), also termed radical nephrectomy for primary lesions, provides a significant survival benefit for patients with mccRCC. Theoretically, removing the primary lesion can effectively reduce the tumor burden and create favorable conditions for subsequent systemic therapy [4–7]. In an era where cytokine therapy has been the only systemic therapy option for patients with mccRCC, a combined treatment regimen of CN and interferon-based adjunctive therapy has shown to be more effective than interferon therapy alone [8, 9]. In the targeted therapy age, several rigorous randomized clinical trials have also demonstrated the promising effectiveness of CN combined with systemic therapy in patients with mccRCC. Owing to the spectacular advantages in relieving local symptoms, such as chronic pain and hematuria, a substantial number of mccRCC patients have received both CN and systemic therapy in the real world [10, 11].

Building a prognostic prediction model is an effective method for identifying the patients who will benefit the most from these treatments. However, a model for predicting the survival of mccRCC patients treated with CN and systemic therapy is still lacking.

As an important subfield of artificial intelligence, machine learning (ML) algorithms involve multiple disciplines and demonstrate advancement compared with traditional tools. Mounting evidence has revealed that ML models can provide a more accurate prognosis prediction by comprehensively integrating and analyzing the complex connections between clinical features and outcomes [12–14]. For example, one of the most prevalent ML algorithms, the gradient boosting machine (GBM), has shown excellent performance in terms of speed and accuracy in both classification and regression models [15]. Nevertheless, the benefit of an ML model in predicting the prognosis of patients with mccRCC receiving both CN and systemic therapy has yet to be fully explored.

**Figure 1.**
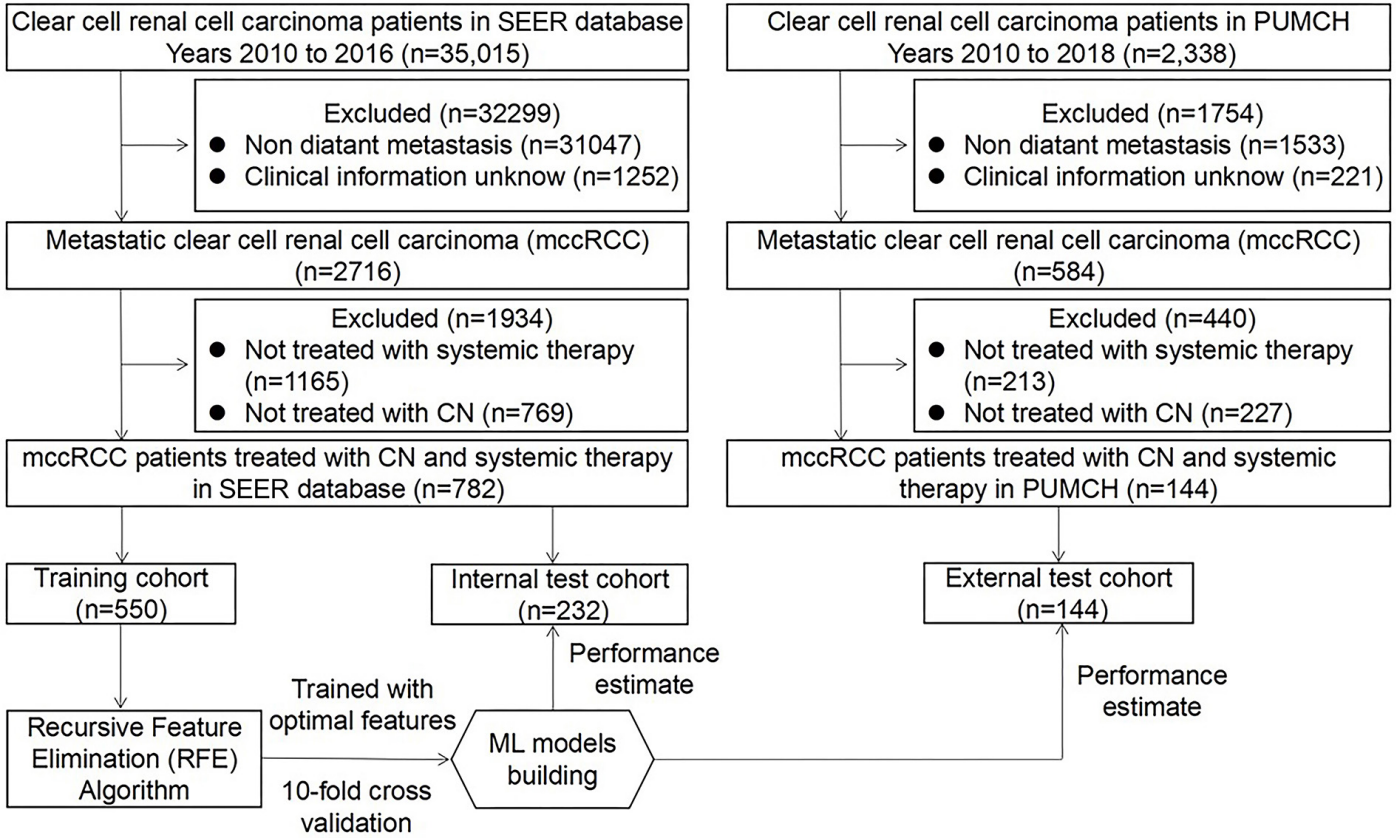
**Strategies for study population selection and establishment of predictive model.** mccRCC: Metastatic clear-cell renal cell carcinoma; ML: Machine learning; CN: Cytoreductive nephrectomy; PUMCH: Peking Union Medical College Hospital.

In this study, we developed several clinical ML models to predict cancer-specific survival (CSS) for patients in this cohort according to data available from the Surveillance, Epidemiology and End Results (SEER) database. Although the specific drug regimens were not available in the SEER database, considering the widespread use of targeted drugs since 2005, systemic therapy in this study mainly refers to angiogenesis therapies and mammalian rapamycin (mTOR) therapies [16]. Furthermore, we used an external test cohort from the Peking Union Medical College Hospital (PUMCH) to test the validity of the models we developed.

## Materials and methods

### Study population

Data concerning mccRCC patients treated with CN combined with systemic therapy were retrospectively extracted from two sources: (I) the SEER database between 2010 and 2016 (https://seer.cancer.gov/) and (II) the PUMCH medical records between 2008 and 2018. The inclusion criteria were as follows: (I) age ≥18 years; (II) confirmation of ccRCC by histology; (III) distant metastases based on the 8th American Joint Committee on Cancer staging systems; (IV) CN treatment; and (V) a history of receiving systemic therapy.

The exclusion criteria were as follows: (I) incomplete information, including unknown age, sex, race, laterality, pathological grade, T stage, N stage, tumor size, metastatic organ sites (bone, brain, liver, and lung), number of metastatic sites, metastasectomy, or radiotherapy; (II) diagnosis of malignant tumors other than ccRCC. The study population selection process is illustrated in Figure 1.

Research involving human participants was reviewed and approved by Peking Union Medical College’s Ethical Committee and Institutional Review Board. All patients have signed an informed consent declaration.

### Outcome and data collection

Considering the relatively high mortality rates of mccRCC patients, we selected CSS as the primary endpoint in this study to avoid discrepancies in deaths. CSS is the interval between the date of treatment and the date of death caused by the tumor. Deaths caused by any factors unrelated to cancer or intervention were identified as non-cancer-specific and censored at the date of death. Patients in the external test (PUMCH) cohort underwent regular physical examinations, laboratory tests, urological ultrasound scans, bone scintigraphy, enhanced computerized tomography (CT) or magnetic resonance imaging every six months. The follow-up was terminated on April 30, 2021.

### ML model establishment and performance evaluation

We employed a recursive feature elimination (RFE) algorithm to select important features for model building. In short, RFE starts with a model that covers all features and gradually removes the features that have the least impact on model performance until the retained features exceed a set performance threshold. The feature subset with the highest accuracy is then selected as the optimal feature combination. To determine the optimal hyperparameters, all the models were trained using 10-fold cross-validation in the training cohort. The data were split into ten parts: one part was assigned to the validation cohort and the other nine parts were used for training. The cross-validation process was repeated ten times, with each part validated once, and the average accuracy of the ten validations gave the final accuracy. Then, four ML algorithms, i.e., the GBM, support vector machine (SVM), random forest (RF), and logistic regression (LR) algorithms, were employed to construct ML models. Receiver operating characteristic (ROC) curves were employed to estimate the ML model accuracy by calculating the area under the curve (AUC). The ROC curve is calculated for all possible cut points (thresholds) and shows the correlation between sensitivity and specificity, thus providing a dynamic and objective response to the model’s performance. Higher AUC values indicated better accuracy of the predictive model. The model fit was evaluated using calibration plots. The decision curve analysis (DCA) method was used to visualize the net benefits and usefulness of the prediction models.

### Statistical analysis

To estimate the differences between groups, categorical variables were expressed as numbers and percentages, and comparisons were made using Chi-square tests or Fisher’s exact tests. The patients were divided into three risk groups: low, intermediate, and high, using the X-tile software (version 3.6.1). All statistical analyses in this study were performed using R software. Statistical significance was defined as *P* < 0.05.

## Results

### Baseline characteristics

This study examined clinical data from 782 patients with mccRCC treated with CN combined with systemic therapy from the SEER database. Overall, 70% (*n* ═ 550) of the patients were randomly divided into the training cohorts, while the remaining 30% (*n* ═ 232) were assigned to the internal test cohort. The median follow-up was 25 (17–31) months in the SEER database. A total of 144 patients were included in the external test (PUMCH) cohort, and the median follow-up for this cohort was 37 (24–52) months. In the SEER cohort, 567 patients (72.5%) had single organ metastases (lung, bone, brain, or liver), 184 (23.5%) had double organ metastases, and 31 (4.0%) had three or more organ metastases. In the PUMCH cohort, 110 patients (76.4%) had single organ metastases, 30 (20.8%) had double organ metastases, and 4 (2.8%) had three or more organ metastases. The lungs were the most common site of metastases at a total of 589 SEER cases (75.3%) and 119 PUMCH cases (82.6%). This was followed by 283 SEER (36.2%) and 43 PUMCH cases (29.9%) of bone metastases, 83 SEER (10.6%) and 13 PUMCH (9.0%) cases of liver metastases, and 74 SEER (9.5%) and 8 PUMCH (5.5%) cases of brain metastases. In addition, 154 SEER (19.7%) and 35 PUMCH (24.3%) patients underwent metastasectomy. Other characteristics of the clinical population and demographics are summarized in Table 1.

**Table 1:** Demographics and clinicopathologic features of the training, internal, and external validation

**Characteristic**		**Training cohort (*n* ═ 550)**	**Internal validation cohort (*n* ═ 232)**	**External validation (PUMCH) cohort (*n* ═ 144)**	***P* value**
Age (years)	<65	362 (65.8%)	160 (69.0%)	86 (59.7%)	0.184
	≥65	188 (34.2%)	72 (31.0%)	58 (40.3%)	
Sex	Male	397 (72.2%)	158 (68.1%)	105 (72.9%)	0.461
	Female	153 (27.8%)	74 (31.9%)	39 (27.1%)	
Race	White	481 (87.5%)	196 (84.5%)	–	<0.001
	Black	27 (4.9%)	14 (6.0%)	–	
	Other	42 (7.6%)	22 (9.5%)	144 (100%)	
Laterality	Left	292 (53.1%)	110 (47.4%)	80 (55.6%)	0.229
	Right	258 (46.9%)	122 (52.6%)	64 (44.4%)	
T stage	T1	47 (8.5%)	25 (10.8%)	9 (6.2%)	0.255
	T2	80 (14.5)	22 (9.5)	15 (10.4)	
	T3	370 (67.3)	158 (68.1)	107 (74.3)	
	T4	53 (9.6%)	27 (11.6%)	13 (9.0%)	
Tumor size (cm)	≤4	22 (4.0%)	6 (2.6%)	1 (0.7%)	0.404
	4~7	108 (19.6%)	52 (22.4%)	33 (22.9%)	
	7~10	188 (34.2%)	85 (36.6%)	49 (34.0%)	
	>10	232 (42.2%)	89 (38.4%)	61 (42.4%)	
N stage	N0	395 (71.8%)	185 (79.7%)	107 (74.3%)	0.069
	N1	155 (28.2%)	47 (20.3%)	37 (25.7%)	
Mets.Lung	No	126 (22.9%)	67 (28.9%)	25 (17.4%)	0.033
	Yes	424 (77.1%)	165 (71.1%)	119 (82.6%)	
Mets.Bone	No	365 (66.4%)	134 (57.8%)	101 (70.1%)	0.024
	Yes	185 (33.6%)	98 (42.2%)	43 (29.9%)	
Mets.Liver	No	500 (90.9%)	199 (85.8%)	131 (91.0%)	0.084
	Yes	50 (9.1%)	33 (14.2%)	13 (9.0%)	
Mets.Brain	No	495 (90.0%)	213 (91.8%)	134 (94.5%)	0.242
	Yes	55 (10.0%)	19 (8.2%)	8 (5.5%)	
Metastatic surgery	Not Performed	445 (80.9%)	183 (78.9%)	109 (75.7%)	0.803
	Performed	105 (19.1%)	49 (21.1%)	35 (24.3%)	
Multiple organ metastasis	Single	407 (74.0%)	160 (69.0%)	110 (76.4%)	0.653
	Two	123 (22.4%)	61 (26.3%)	30 (20.8%)	
	Three-Four	20 (3.6%)	11 (4.7%)	4 (2.8%)	
Histological grade	I-II	91 (16.5%)	38 (16.4%)	24 (16.7%)	0.997
	III-IV	459 (83.5%)	194 (83.6%)	120 (83.3%)	
Radiotherapy	No	376 (68.4%)	150 (64.7%)	105 (72.9%)	0.244
	Yes	174 (31.6%)	82 (35.3%)	39 (27.1%)	

PUMCH: Peking Union Medical College Hospital; Mets: Metastasis organ site.

### Feature selection

**Figure S1.**
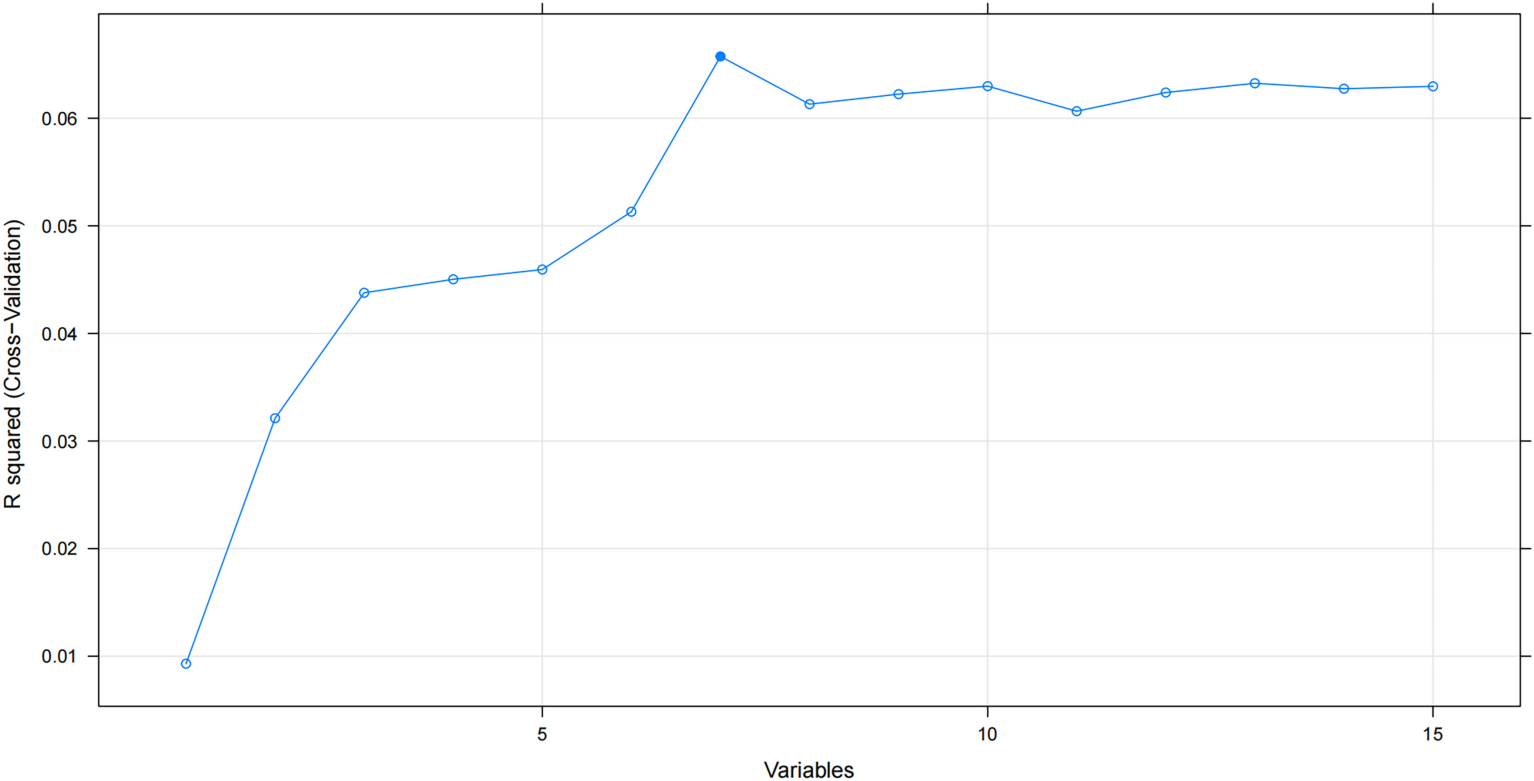
The correlation between included features numbers and the prediction fitness in the recursive feature elimination (RFE) algorithm.

Incorporating redundant features may degrade the performance of an ML model [17]. The RFE algorithm was employed as a feature selection method to identify the optimal feature subset among all features. After RFE screening, seven important features, namely, pathological grade, T stage, N stage, the number of metastatic sites, brain or liver metastases, and metastasectomy, were determined. These features were then included in all our ML models in both the training and testing cohorts (Figure S1).

### ML models accurately predicted patient prognosis

**Figure 2.**
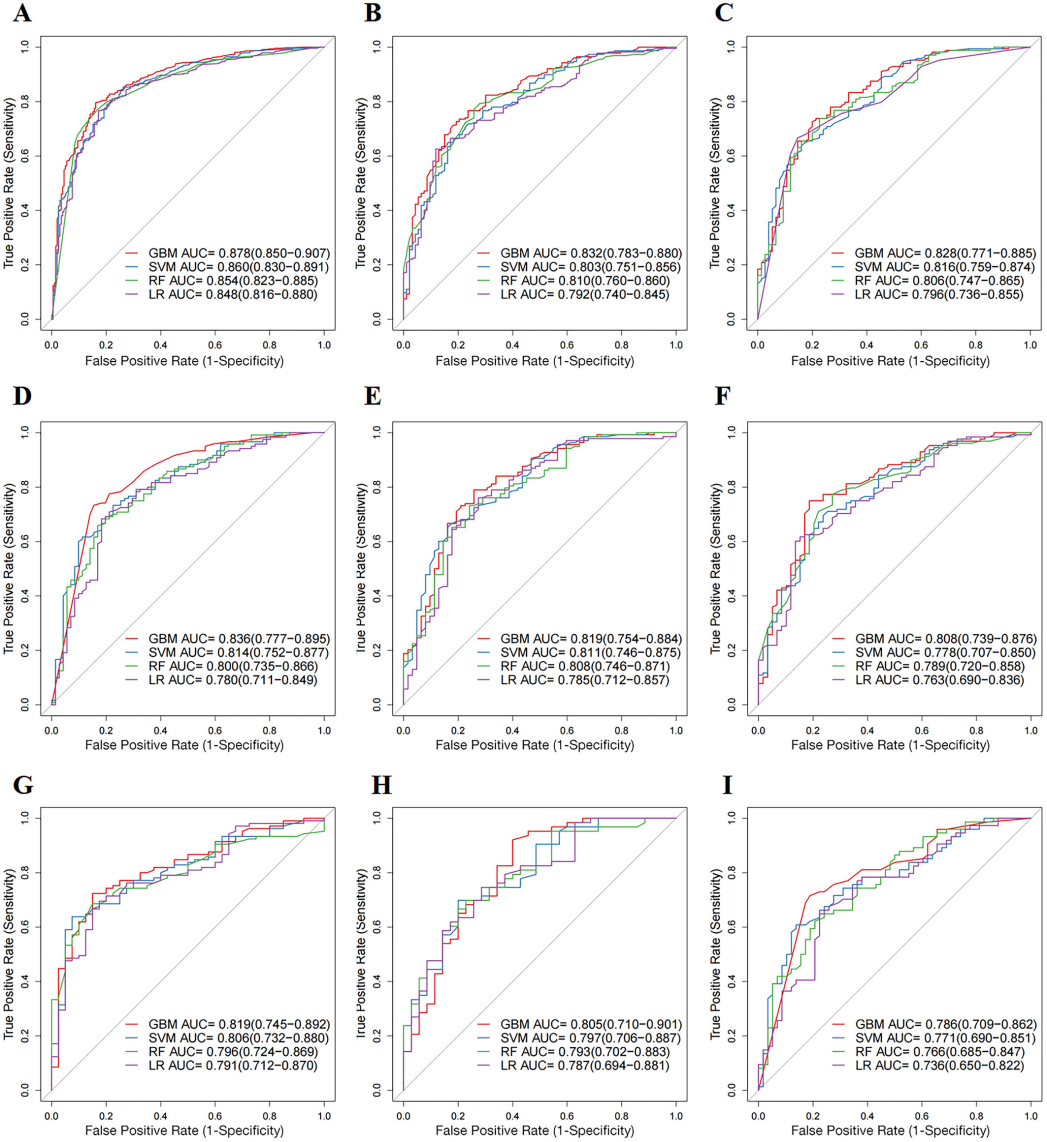
**Receiver operating characteristic (ROC) curves of four ML models (GBM, SVM, RF, and LR).** (A)–(C) AUC values of predictive models at 1-, 3-, and 5-year CSS in the training cohorts, respectively; (D)–(F) AUC values of predictive models at 1-, 3-, and 5-year CSS in the internal validation cohorts, respectively; (G)–(I) AUC values of predictive models at 1-, 3-, and 5-year CSS in the external validation cohorts, respectively. ML: Machine learning; GBM: Gradient boosting machine; SVM: Support vector machine; RF: Random forest; LR: Logistic regression; AUC: Area under the receiver operating characteristic curves.

**Figure 3.**
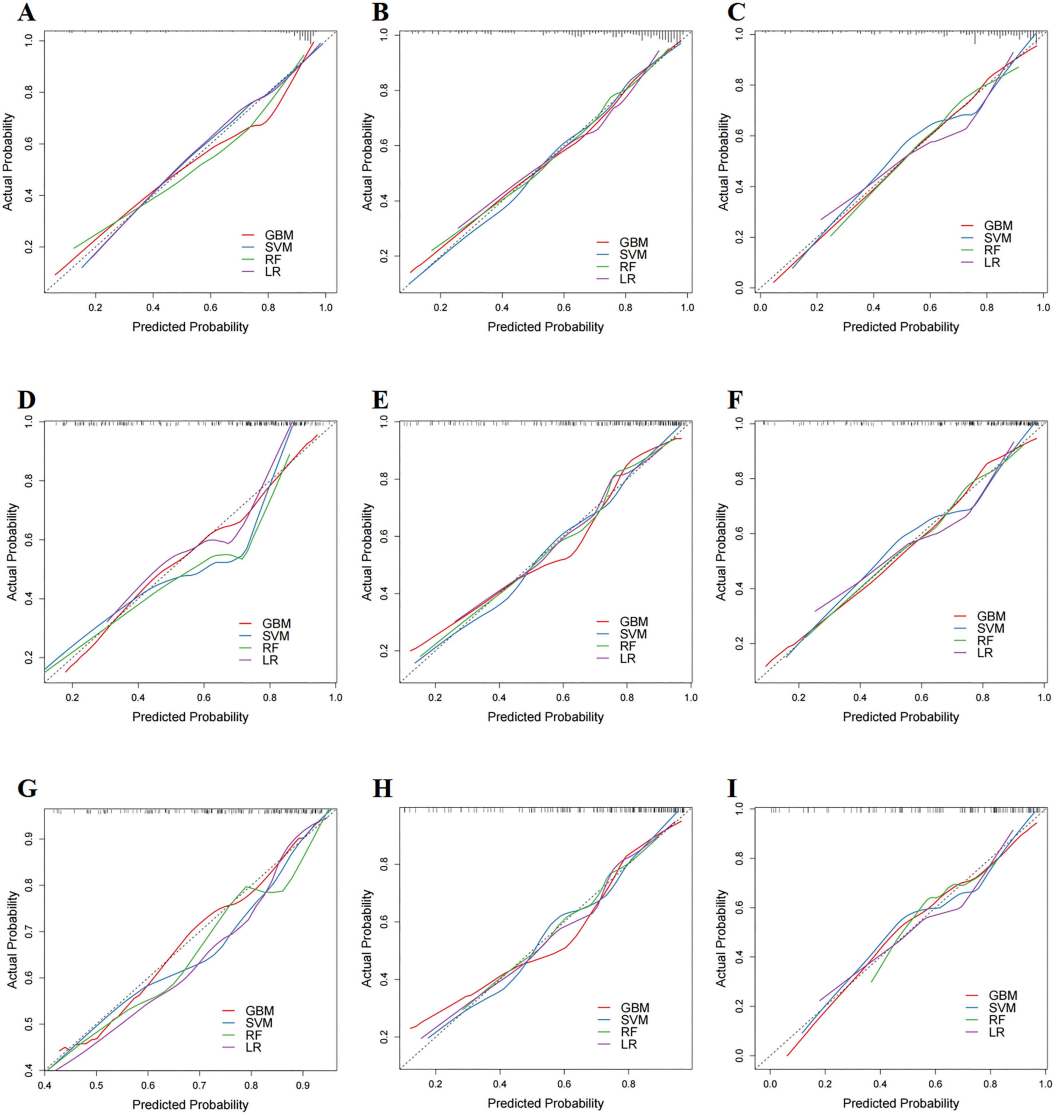
**Calibration curves for predicting CSS of patients with four ML models (GBM, SVM, RF, and LR) at 1, 3, and 5 years, respectively.** (A)–(C) The calibration curves of predictive models in the training cohorts; (D)–(F) The calibration curves of predictive models in the internal validation cohorts; (G)–(I) The calibration curves of predictive models in the external validation cohorts. CSS: Cancer-specific survival rate; ML: Machine learning; GBM: Gradient boosting machine; SVM: Support vector machine; RF: Random forest; LR: Logistic regression.

We established prognosis prediction models for mccRCC patients treated with CN and systemic therapy using four ML algorithms (GBM, SVM, RF, and LR). To evaluate the discriminatory abilities of these models, ROC curves for 1-, 3-, and 5-year CSS were constructed. In the training cohort, the AUC values of these ML models for the prediction of 1-, 3-, and 5-year CSS were 0.878, 0.832, and 0.828 for GBM; 0.860, 0.803, and 0.816 for SVM; 0.854, 0.810, and 0.806 for RF; and 0.848, 0.792, and 0.796 for LR, respectively. Compared with the other three ML models, the GBM model had the highest accuracy in predicting 1-, 3-, and 5-year CSS. Similar results were found in the internal and external test cohorts (Figure 2A–2C). In the internal test cohort, the AUC values of the GBM model for the prediction of 1-, 3-, and 5-year CSS were 0.836, 0.819, and 0.808, respectively (Figure 2D–2F). In the external test cohort, the AUC values of the GBM model for the prediction of 1-, 3-, and 5-year CSS were 0.819, 0.805, and 0.786, respectively (Figure 2G–2I). In addition, calibration curves for the four ML models are demonstrated in Figure 3. Compared to the other three ML models, the calibration curves of the GBM model were the closest to the ideal lines.

### Clinical value of the ML models

**Figure 4.**
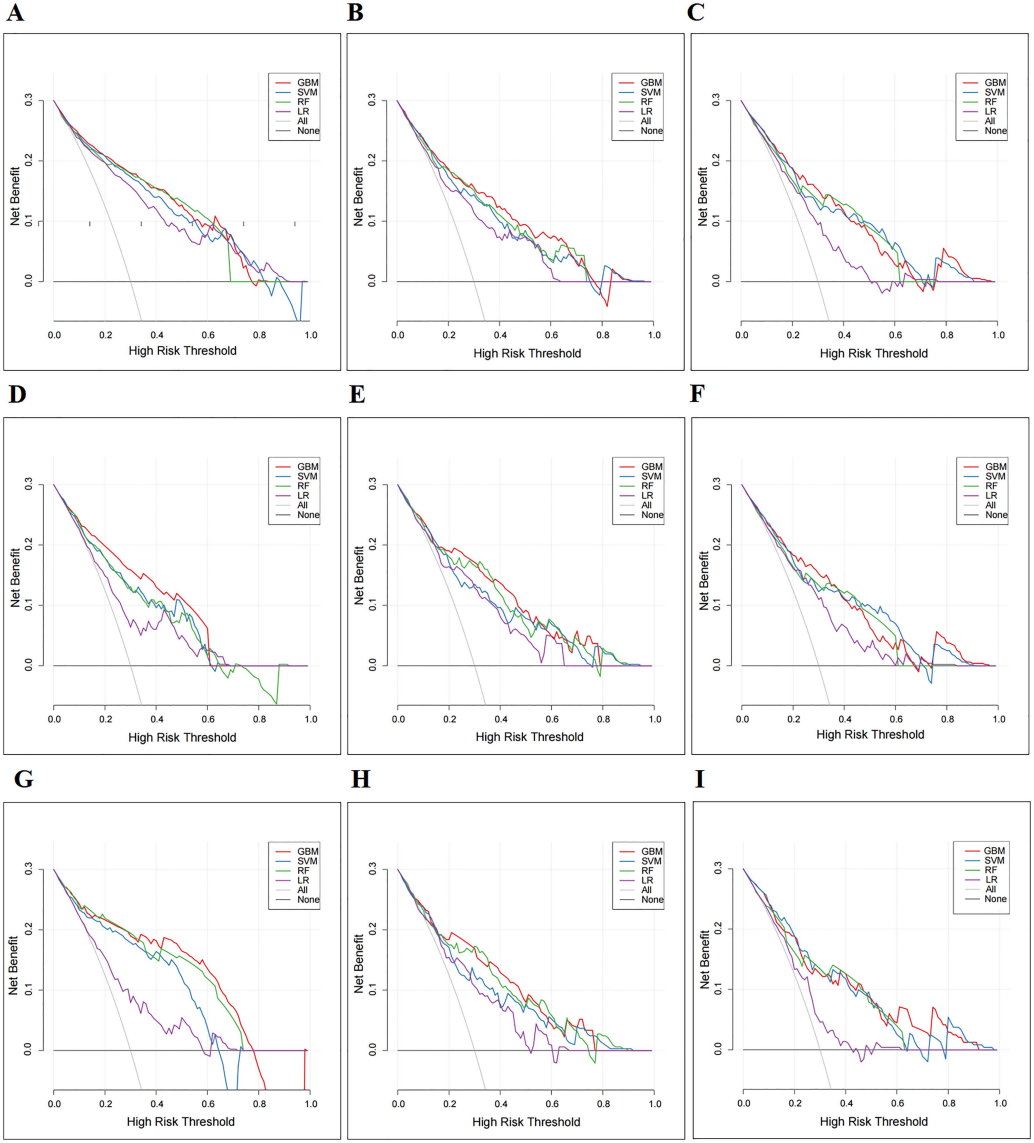
**Decision curve analysis demonstrating the clinical benefit of four ML models (GBM, SVM, RF, and LR).** (A)–(C) Training cohorts; (D)–(F) Internal validation cohorts; (G)–(I) External validation cohorts. ML: Machine learning; GBM: Gradient boosting machine; SVM: Support vector machine; RF: Random forest; LR: Logistic regression.

**Figure S2.**
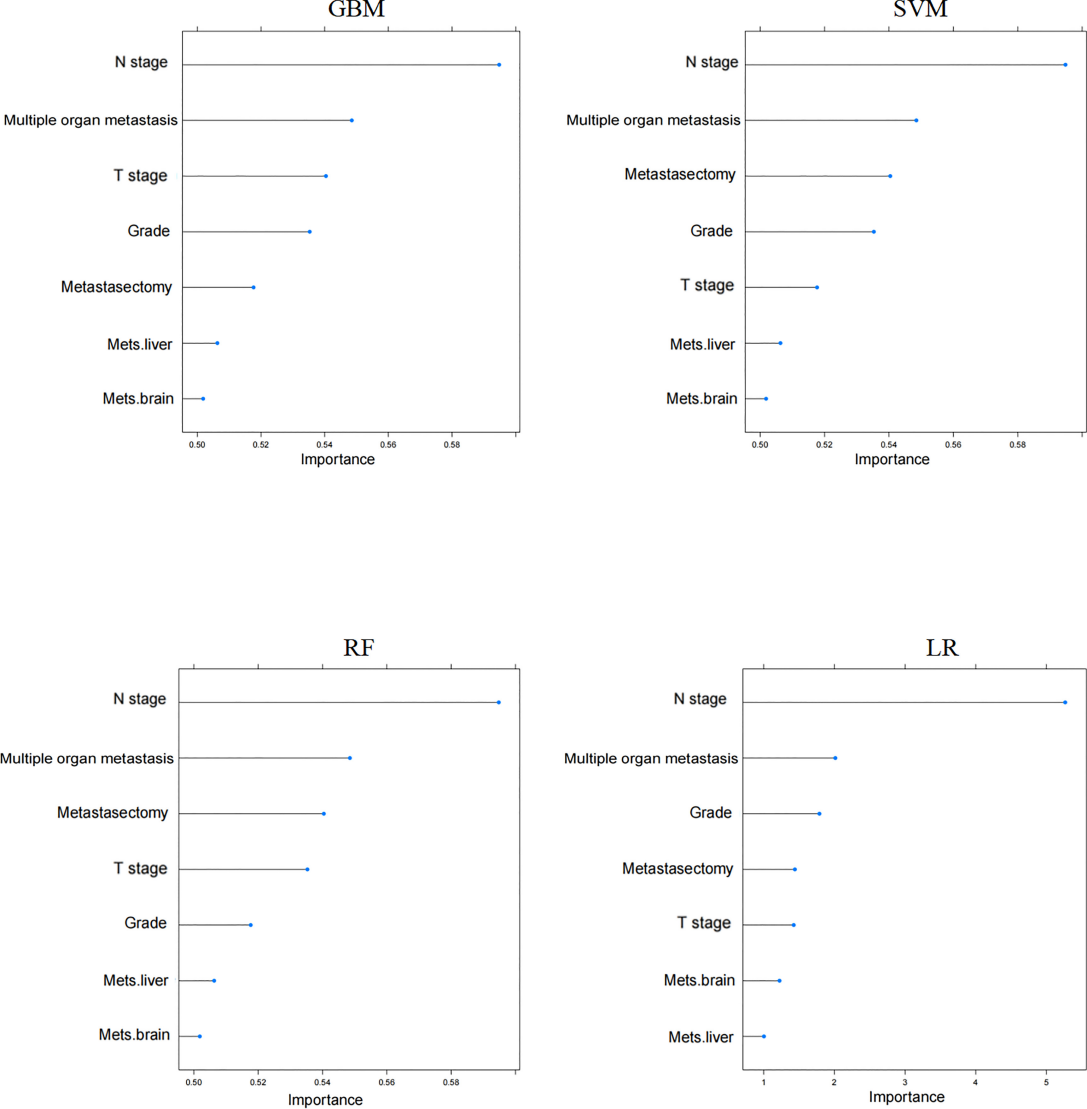
**The relative importance of features in four ML models (GBM, SVM, RF, and LR).** GBM: Gradient boosting machine; SVM: Support vector machine; RF: Random forest; LR: Logistic regression; Mets: Metastasis organ site.

DCA is a novel method for visualizing whether the use of a prediction model in clinical practice will benefit decision-making. The percentage of threshold probability is displayed on the *X*-axis, and the net benefit is indicated on the *Y*-axis [18, 19]. In our study, we hypothesized that patients whose predicted probability exceeds a set threshold would benefit from CN combined with systemic therapy. DCA indicated that all ML models achieved a net benefit. The DCA of the GBM has higher net benefits in the majority of the cohort subgroups, indicating that it had better clinical outcome values (Figure 4). Finally, the varying importance of the features for predicting CSS in each ML model is shown in Figure S2.

### Risk stratification

**Figure 5.**
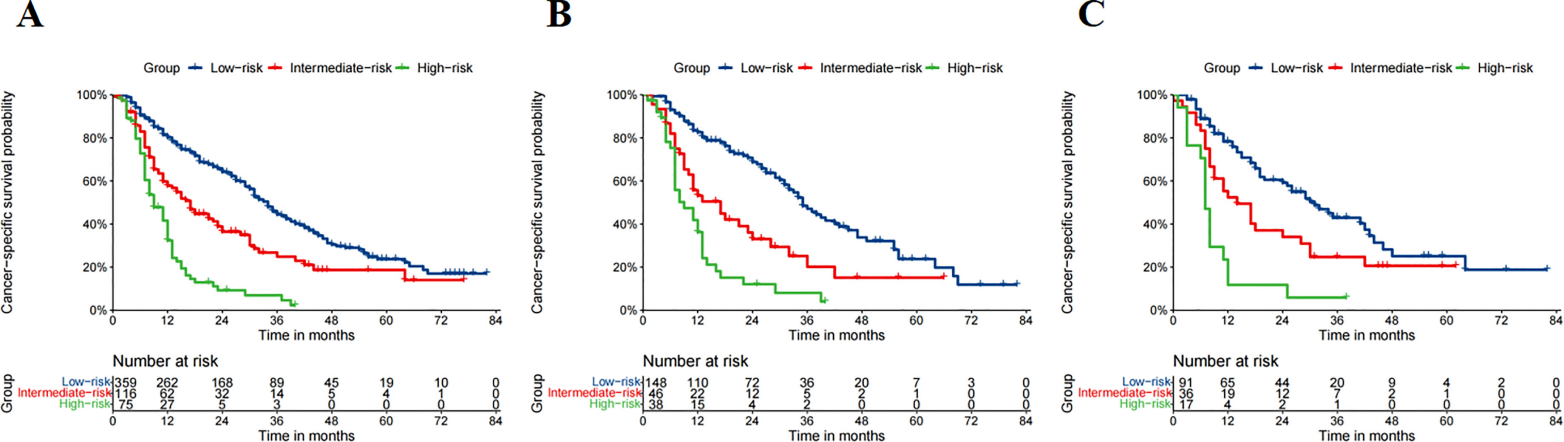
**Kaplan–Meier showing disparities between groups.** (A) CSS stratified by GBM model in the training cohorts; (B) CSS stratified by GBM model in the internal validation cohorts; (C) CSS stratified by GBM model in the external validation cohorts. CSS: Cancer-specific survival rate; GBM: Gradient boosting machine.

**Figure S3.**
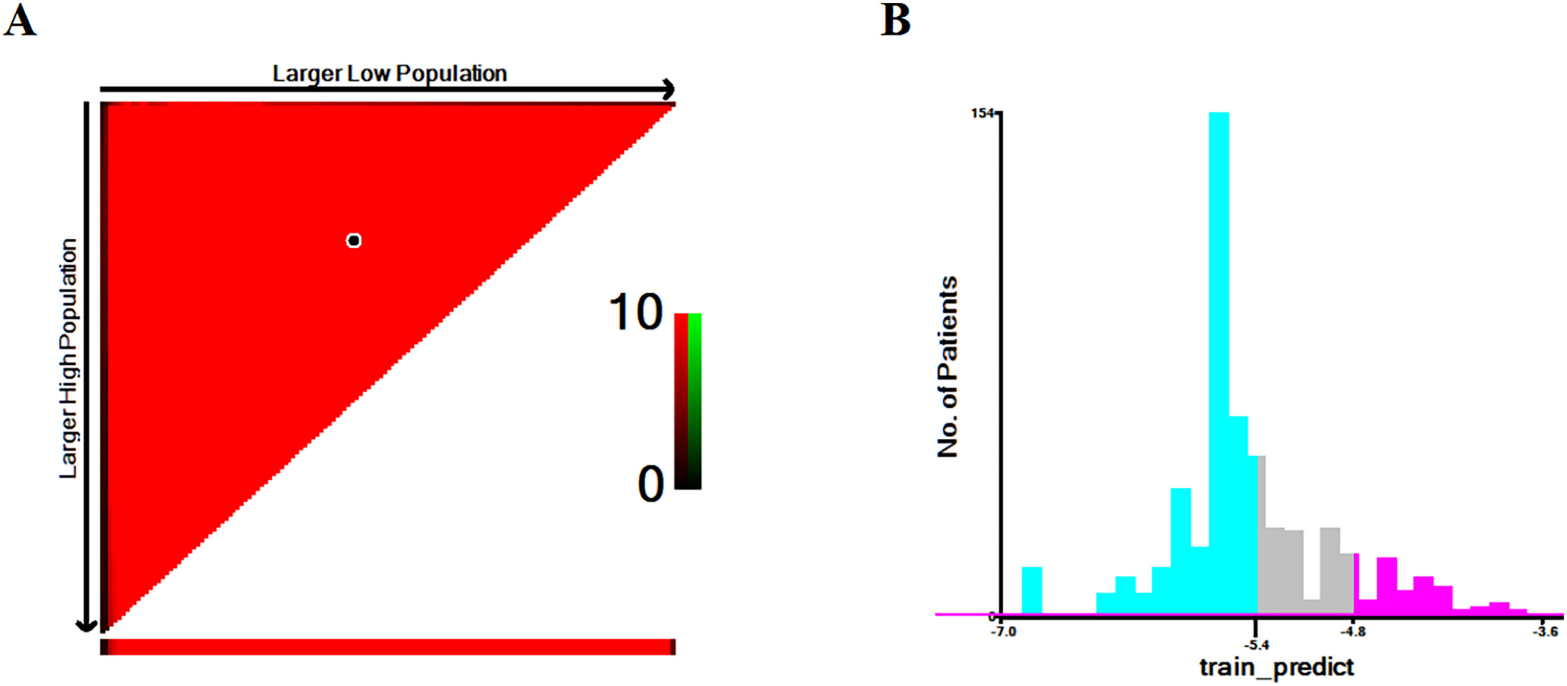
**Determine the optimal cutoffs for GBM via X-tile analysis.** (A) The optimal cutoffs highlighted by black circle; (B) Numbers of patients in high, intermediate, and low mortality risk subsets. GBM: Gradient boosting machine.

As GBM was the optimal ML model based on the performance evaluation above, we set two optimal cut-off values (−5.4 and −4.8) depending on the GBM prediction score and divided patients into high-, intermediate-, and low-risk groups via X-tile analysis (Figure S3). In the training cohort, the long-term survival (5-year CSS) of each group was as follows: high-risk, 2.3%; intermediate-risk, 18.7%; and low-risk, 23.7%. We then verified the actual performance of the aforementioned three risk strata in the internal and external test cohorts. In the internal test cohort, the 5-year CSS was 4.0% in the high-risk group, 15.1% in the intermediate-risk group, and 26.2% in the low-risk group. In the PUMCH cohort, the 5-year CSS was 5.9% in the high-risk group, 21.2% in the intermediate-risk group, and 22.8% in the low-risk group (Table 2). According to these results, the ML model provided excellent prognostic stratification in the training, internal test, and external test cohorts. The survival curves of patients with different risk stratifications also showed that the GBM model provided excellent prognostic stratification (Figure 5A–5C).

**Table 2:** Cancer-specific survival (CSS) according to risk stratification

**Risk group**	**1-year CSS, % (95% CI)**	**3-year CSS, % (95% CI)**	**5-year CSS, % (95% CI)**	**Hazard ratio % (95% CI)**	***P* value**
Training cohorts (*n* ═ 550)					
Low-risk	80.2 (76.0–84.5)	44.4 (38.7–51.1)	23.7 (18.0–31.2)	Ref	
Intermediate-risk	57.7 (49.1–67.9)	24.9 (16.9–36.7)	18.7 (11.3–31.1)	1.82 (1.33–2.48)	<0.001*
High-risk	32.4 (23.0–45.6)	6.9 (2.6–18.3)	2.3 (0.4–15)	2.02 (1.41–2.90)	<0.001^✝^
Internal validation cohorts (*n* ═ 232)					
Low-risk	83.1 (77.2–89.5)	48.4 (39.6–59.3)	26.2 (17.6–38.9)	Ref	
Intermediate-risk	53.1 (40.0–70.6)	20.0 (9.6–42.2)	15.1 (6.0–38.3)	2.24 (1.33–3.80)	<0.001*
High-risk	36.3 (23.5–55.9)	8.1 (2.4–27.1)	4.0 (0.6–25.4)	1.79 (1.08–2.96)	<0.001^✝^
External validation cohorts (*n* ═ 144)					
Low-risk	78.8 (70.8–87.8)	45.8 (34.6–57.9)	22.8 (13.2–39.3)	Ref	
Intermediate-risk	52.4 (38.3–71.7)	25.2 (15.4–46.2)	21.2 (10.9–41.2)	1.72 (1.02–2.91)	<0.001*
High-risk	11.8 (3.2–43.2)	5.9 (0.9–39.4)	5.9 (0.9–39.4)	2.14 (1.03–4.41)	<0.001^✝^

*P value versus low-risk; ✝*P* value versus intermediate-risk.

## Discussion

At present, positive associations between cytoreductive resection of primary tumors and superior overall survival rates have been demonstrated in a variety of solid metastatic tumors, such as advanced-stage endometrial cancer, cohesive gastric cancer, and ovarian cancer [20–22]. As for mccRCC, a significant survival benefit associated with CN and systemic therapy was found in a study by Chakiryan et al., who constructed an analysis containing 5005 mccRCC patients using the National Cancer Database registry data as the instrumental variable. Similar results were also reported by Zhang et al. and McIntosh et al. based on clinical data from the SEER database [23–25].

However, given the potential surgical complications and toxic effects of systemic therapy, a corresponding prognosis prediction model needs to be developed to improve patient selection and outcome prediction. Several prognostic prediction models have been used for mccRCC patients, such as the International Metastatic Renal Cell Carcinoma Database Consortium model and the Memorial Sloan-Kettering Cancer Center model. However, models that specialize in predicting the prognosis of mccRCC patients receiving both CN and systemic therapy, especially ML models, have yet to be developed [26, 27].

Therefore, we aimed to develop a practical survival prediction model to accurately predict the individualized survival of mccRCC patients using ML algorithms. In this study, we established four ML models to predict the prognosis of these patients. Among them, GBM was the best model in terms of accuracy, fitness, and clinical application. To our knowledge, this is the first study to apply ML algorithms to predict mccRCC patient survival in such a large patient cohort.

One of the most significant advantages of ML models over traditional predictive models is their ability to analyze feature importance and provide optimal feature subsets without requiring manual processing, resulting in models with high accuracy and stability. To date, multiple ML models have been developed and validated for predicting the prognosis of ccRCC patients based on medical imaging, gene expression data, or clinical information. For instance, Nazari et al. constructed a radiomics-based predictor using several ML algorithms to analyze CT images, which could accurately predict the 5-year survival of ccRCC patients [28]. However, a specific ML model for predicting the prognosis of patients with mccRCC remains undeveloped.

In our study, the clinical data of over 900 mccRCC patients treated with CN and systemic therapy were included. We then constructed four ML models (GBM, SVM, RF, and LR) for 1-, 3-, and 5-year CSS prediction. Model performance was evaluated using ROC curves, calibration plots, and DCA. In the training, internal validation, and external validation cohorts, the GBM model demonstrated the highest level of prediction accuracy and more favorable correlations. Furthermore, according to the DCA results, the GBM model can effectively assess the advantages and disadvantages of clinical decisions. In clinical research, GBM models are gaining increasing traction. We are the first to apply the GBM model to mccRCC survival prediction.

Selecting the most effective features from the original variables to reduce the dimensionality of the datasets is a key step in improving the performance of an ML model [17]. Using RFE methods, seven features, including pathological grade, T stage, N stage, number of metastatic sites, brain or liver metastases, and metastasectomy, were selected, and the optimal feature subset was chosen for further analysis. In the next step, the relative importance of each input feature was ranked using ML models. Despite the slight differences in feature importance ranking, histological grade, tumor stage, lymph node stage, number of metastatic sites, and metastasectomy were ranked in the top five in all ML models. These results revealed that mccRCC patients with lower histological grade, earlier tumor stage, no indication of lymph node metastases, fewer metastatic sites, and who received metastasectomy may have better prognoses after treatment with CN and systemic therapy.

Previous studies have also indicated that radiotherapy is significantly associated with better prognosis in patients with mccRCC [29, 30]. However, in this study, radiotherapy was not selected by the RFE algorithm for the optimal feature subset. Further studies on the influence of radiotherapy on the prognosis of patients with mccRCC treated with CN and systemic therapy are needed.

There have been dramatic changes in the treatment of mccRCC over the past few decades. The role of CN has become increasingly unclear with the advent of new treatment options. According to the results of the CARMENA trial and SURTIME trials, in comparison with the CN, sunitinib alone did not show any inferiority. However, all of the above studies were deemed to be underpowered, limited by insufficient study subjects, slow to accrue and lack of homogeneity in patients selection. Based on these limitations, it is important to select patients appropriately and publish prospective studies that contain high levels of evidence [31].

After the era of cytokine and targeted therapies, mccRCC treatment has gradually entered the era of immunotherapy. Nirmish et al. compared the prognosis of mccRCC patients treated with immune checkpoint inhibitors (ICIs) alone or CN combined with ICIs. The result showed that CN plus immunotherapy had a longer OS than immunotherapy alone based on the NCDB datasets [32]. Thus, despite the uncertainty of efficacy, CN remains an important treatment option, especially for alleviating hematuria or pain. However, optimal candidates for CN need to be carefully screened. Recent perspectives supported that CN could be performed in a patient with a kidney that is in place and a disease of favorable or intermediate risk [33].

In this study, the GBM model identified approximately 15% of patients with mccRCC in the high-risk group who experienced an extremely poor 5-year survival rate after receiving CN and systemic therapy. Conversely, more than half of the mccRCC patients were classified as low-risk patients. The low-risk subset displayed relatively satisfactory long-term CSS, indicating that CN combined with systemic therapy is particularly suitable for this population. With the above risk stratification, overtreatment can be largely avoided.

Present study has several limitations: First, because the training set data used for building models were from the SEER database, the treatment regimen and information on systemic therapy were not available. The latest studies have reported that deferred CN may be more beneficial for patients who respond favorably to systemic therapy compared with upfront CN [6, 34, 35]. Second, the SEER database lacks data on some vital clinical characteristics, such as basic diseases, surgical complications, and biochemical indicators, which may influence the accuracy of the model prediction. Third, there is inter-group heterogeneity in the external validation cohort compared with the data from the SEER database, which may be related to the relatively small amount of data in the external validation cohort. Therefore, a multi-center study must be conducted to further validate the model performance.

Despite mentioned limitations, these ML models, built on a large population database and validated with data from external groups, provide the first targeted and practical survival prediction tools for patients with mccRCC receiving both CN and systemic therapy, which have a high potential for use in clinical practice.

## Conclusion

We developed and validated four ML models based on significant clinicopathological characteristics for predicting CSS in patients with mccRCC treated with CN and systemic therapy. These models will not only be used in CSS prediction, patient risk stratification, and clinical decision making but also encourage further research on the use of ML algorithms to improve personalized prognostic prediction.

## Supplemental Data
